# Infodemic Source Detection with Information Flow: Foundations and Scalable Computation †

**DOI:** 10.3390/e27090936

**Published:** 2025-09-06

**Authors:** Zimeng Wang, Chao Zhao, Qiaoqiao Zhou, Chee Wei Tan, Chung Chan

**Affiliations:** 1 Department of Computer Science, City University of Hong Kong, Hong Kong, China; zimenwang4-c@my.cityu.edu.hk (Z.W.); ao.ao@my.cityu.edu.hk (C.Z.); 2 Faculty of Computer Science and Control Engineering, Shenzhen University of Advanced Technology, Shenzhen 518055, China; zhouqiaoqiao@suat-sz.edu.cn; 3 College of Computing and Data Science, Nanyang Technological University, Singapore 639978, Singapore

**Keywords:** infodemic source detection, information flow, submodular optimization

## Abstract

We consider the problem of identifying the source of a rumor in a network, given only a snapshot observation of infected nodes after the rumor has spread. Classical approaches, such as the maximum likelihood (ML) and joint maximum likelihood (JML) estimators based on the conventional Susceptible–Infectious (SI) model, exhibit degeneracy, failing to uniquely identify the source even in simple network structures. To address these limitations, we propose a generalized estimator that incorporates independent random observation times. To capture the structure of information flow beyond graphs, our formulations consider rate constraints on the rumor and the multicast capacities for cyclic polylinking networks. Furthermore, we develop forward elimination and backward search algorithms for rate-constrained source detection and validate their effectiveness and scalability through comprehensive simulations. Our study establishes a rigorous and scalable foundation on the infodemic source detection.

## 1. Introduction

Identifying the source of a contagion is a critical task across multiple fields, including cybersecurity [1,2], public health [3,4], and social networks [5,6,7,8,9]. The rapid dissemination of misinformation and malicious content can have severe consequences, such as distorting public perception [10], inciting panic [11], and compromising digital infrastructure [12]. Therefore, the ability to accurately and efficiently pinpoint the original source of a contagion within a network becomes essential. This task typically involves analyzing a static snapshot of the network, referred to as the contagion graph, which illustrates the relationships among nodes classified as “infected” or “susceptible” according to their exposure status. The conceptual foundation for modeling contagion dynamics in networks is frequently built upon the classical Susceptible–Infectious (SI) model put forth by Bailey [13].

The task of detecting the source of the contagion was pioneered by [14] as a maximum likelihood (ML) estimation within the framework of the SI model with exponential spreading time for regular trees. Their method maximizes the likelihood of observing the state of infection by an initial source node. Then, the rumor center can be determined using message-passing algorithm [15]. They extended the framework by introducing the rumor centrality as the estimator, where nodes are ranked according to the likelihood that they were original sources of the contagion. Shah and Zaman [16] broadened the graph structure to irregular trees and overcame the limitation of the exponential spreading time by multi-type continuous time branching processes/generalized Polya’s urn models [17]. The rumor source detection problem over the SI model was then extended to degree-regular graph with multiple cycles by a backward contact tracing method [18,19]. Fan and Wang [20] extended the problem to continuous spreading time with probabilistic detection algorithms and proposed the joint maximum likelihood (JML) estimator, which incorporates both infected nodes and spreading time. Moreover, they demonstrated that the JML estimator can be computed with a computational complexity of O(nLlogL) by utilizing the message-passing algorithm, where *n* is the number of infected nodes, and *L* is the effective range of the spreading time.

Although these graph-based approaches provide computationally efficient estimators, we observe that they may fail to correctly identify the source, even in a fundamentally simple graph structure, as explained in the next section. To address this limitation, it is essential to move beyond graphs and leverage the theory of information flow. Network coding theory established that mixing information at intermediate nodes achieves the multicast rate [21], and linear coding was shown to be sufficient for this purpose [22]. An algebraic formulation provided a systematic framework for constructing such codes [23], and polynomial-time deterministic algorithms were later developed for constructing capacity-achieving multicast codes [24]. Efficient algorithms were developed to compute coding solutions while maintaining the optimality guaranteed by classical max-flow bounds [25], whereas random linear network coding enabled capacity-achieving multicast in a fully distributed and probabilistic manner [26]. In parallel, submodular optimization emerged as a powerful tool for characterizing network information flow. The theory of submodular flows [27] and information-theoretic formulations of entropy and information inequalities [28,29] established a unified view of rate-constrained communication. These results motivate our generalization of source detection beyond graph-based contagion models, leading to a rate-constrained formulation grounded in information flow.

This work extends our previous conference paper [30]. The journal version introduces a new lazy–greedy forward search algorithm that improves the computational efficiency of rate-constrained source detection. Furthermore, we provide extensive new experiments on synthetic network models to demonstrate the scalability and the achieved multicast rate. For the sake of clarity, completeness, and readability, we explicitly restate the fundamental definitions, lemmas, propositions, and selected examples from our earlier paper throughout this article, including full proofs that were omitted in the concise five-page conference version.

The structure of this article is organized as follows: In Section 2, we re-define the SI model and the ML/JML estimators and reveal the limitation of conventional ML estimation methods. Section 3 generalizes the source detection problem with network flow and introduces the rate-constrained formulation. Section 4 proposes our main results on algorithmic solutions based on backward elimination and greedy forward search. We also present experimental results validating the effectiveness and scalability of the proposed methods. We discuss implications and future directions in Section 5 and conclude the paper in Section 6.

## 2. Preliminary

We first provide a brief overview of the two estimation methods, ML and JML, along with their formulations. Given a finite undirected graph G=(V,E) with vertex set *V* and edge set E⊆V2, at time t=0, an initial set of infected nodes W⊆V is specified. For every ordered pair (u,v) with {u,v}∈E, there is an independent random time $\tau_{uv}∼\mathrm{Exp}\left(\lambda \right)$, where Exp(λ) is the exponential distribution with mean 1/λ. Let $T_{u}$ denote the time at which node *u* first becomes infectious. For u∈W, set $T_{u}=0$. Recursively, for each susceptible *v*,

$$T_{v}=\min_{\begin{array}{c} u:\{u,v\}\in E\\ T_{u}<\infty \end{array}}\left[T_{u}+\tau_{uv}\right].$$

For any t≥0, the infected set is

$$W\left(t\right):=\left\{u\in V:T_{u}\leq t\right\}.$$

Now, given a non-empty subset S⊆Vk of k>0 nodes which are currently infected, the ML estimator selects a source node as the solution to

$$\max_{s\in S}P\left[W^{\left(k\right)}=S|W^{\left(1\right)}=\left\{s\right\}\right],$$

where $W^{\left(k\right)}$ is a random set denoting the set of first *k* infected nodes including the source node. The JML estimator selects a source node as the solution to

$$\max_{s\in S}\underset{t\geq 0}{\sup}P\left[W\left(t\right)=S|W\left(0\right)=\left\{s\right\}\right],$$

where W(t) is a random set denoting the set of all infected nodes by time t≥0, assuming the source node is infected at time 0. Different from the ML estimator, the JML estimator further optimizes over the time *t* when *S* is observed.

**Figure 1:**
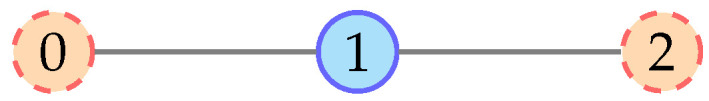
Example network where node 1 is the rumor source node and both nodes 0 and 2 are infected. Source node is in cyan. Orange dots with dashed red border represent infected nodes.

We show that, in Figure 1, the ML estimation score is 1 when 0 is the source, which corresponds to the infection sequence (0,1,2). If the infection sequence is (1,2,0) and (1,0,2), and node 1 is the source, the ML estimation score is $\frac{1}{2}\times 1+\frac{1}{2}\times 1=1$, which is exactly the same as the case for node 0. By symmetry, node 2 has the same chance of being the source as node 0 and, consequently, node 1. Such equal chances cause the source detection problem to become degenerate. Similarly for JML, the method also suffers from the same degeneracy because, for connected graphs, all nodes will eventually be infected, i.e., the JML estimation score increases to 1 as t→∞ regardless of the choice of the source node. The likelihood probability when the set of infected nodes at time 0 is {1} is

$$\begin{array}{c} P\left[W\left(t\right)=\left\{0,1,2\right\}|W\left(0\right)=\left\{1\right\}\right]=P[T_{0}\left(\left\{1\right\}\right)\leq t,T_{2}\left(\left\{0,1\right\}\right)\leq t], \end{array}$$

where $T_{u}\left(B\right)$ is the time set *B* infects node *u*. By symmetry, we consider the case when W(0)={0} and achieve the following

$$\begin{array}{c} P\left[W\left(t\right)=\left\{0,1,2\right\}|W\left(0\right)=\left\{0\right\}\right]=P[T_{0}\left(\left\{1\right\}\right)\leq t,T_{2}\left(\left\{0,1\right\}\right)\leq t-T_{0}\left(\left\{1\right\}\right)]. \end{array}$$

When considering the observation time *t* that achieves the supremum in the JML estimator, i.e., t→∞, the JML estimation scores for both nodes 0 and 1 converge to 1, leading to a degeneracy.

Beyond the previously discussed degeneracy issues in ML and JML estimators, conventional graphical models also exhibit fundamental limitations in capturing the actual structure of information flow in networks. Specifically, they fail to account for the rate at which information spreads: if a set *B* is currently infected, the infection rate of a node u∈V∖B should be proportional to the capacity of a channel from *B* to *u*. Moreover, since a rumor can be viewed as a piece of information that spreads through the network, it is natural to characterize its spreading by a transmission rate. Suppose that the rumor is observed to reach a subset of nodes A⊆V at a rate of at least r≥0. Any candidate source set S⊆V must then be able to deliver information to *A* at no less than this rate. Or, the network multicast capacity must be no less than this rate. Such rate constraints are not considered in the existing model.

### 2.1. SI Model

To overcome these limitations, we re-define the SI model and improve the formulation of the estimator. To capture information flow in a network consisting of a discrete set *V* of nodes, we generalize the SI model using the following infection rate tuple $\lambda_{V}:=\left(\lambda_{u}|u\in V\right)$, where $\lambda_{u}:2^{V}\to R_{+}$, where

(1) $$\begin{array}{cccc} \lambda_{u}\left(\emptyset \right) & =0\;\mathrm{and}\;\lambda_{u}\left(B^{\prime }\right)\leq \lambda_{u}\left(B\right) & \; & \forall B^{\prime }\subseteq B\subseteq V. \end{array}$$

A more elaborated model will be given in Definition 5.

To define the SI model for infodemic, define the time for u∈V to be infected by B⊆V∖{u} by the exponentially distributed random variables

(2) $$T_{u}\left(B\right)∼\mathrm{Exp}\left(\lambda_{u}\left(B\right)\right)$$

mutually independent over *u* and *B*. Let U(t) be the sequence of infected nodes at time t≥0, i.e., $U\left(t\right)=\left(u_{1},\ldots ,u_{k}\right)$ means $u_{i}$ is the *i*-th infected node by time *t* for i∈[k]:={1,…,k}. For notational convenience, let $u^{k}=\left(u_{1},\ldots ,u_{k}\right)$ and $u_{i}^{j}=\left(u_{i},\ldots ,u_{j}\right)$ for i≤j. Then, the infection times determine the sequence of infected node as follows:

**Definition** **1** (SI)**.** *Given $U\left(\tau \right)=u^{k}$, for all t>τ,*

(3) $$U\left(t\right)=u^{k+1}\Leftrightarrow u_{k+1}=\arg\min_{w\in V\setminus \mathrm{set}(u^{k})}T_{w}\left(\mathrm{set}\left(u^{k}\right)\right)$$

*where the set function*

(4) $$\mathrm{set}\left(u^{k}\right):={\left\{u_{i}\right\}}_{i=1}^{k}$$

*turns the input sequence $u^{k}$ into an unordered set.*

A unique choice of $u_{k+1}$ is possible because, almost surely, $T_{w}\left(\mathrm{set}\left(u^{k}\right)\right)$s are distinct.

The source detection problem is to find set(U(0)) given $\mathrm{set}(U\left(T^{\left(k\right)}\right))=S$, where

(5) $$T^{\left(k\right)}=\inf\left\{t\geq 0|\;|\mathrm{set}(U(t))|=k\right\},$$

namely, the time to infect the first *k* nodes.

We propose the following source detection problem for the SI model:

**Definition** **2** (ML for independent observation time)**.** *The likelihood probability of observing the set S of infected nodes at time t≥0, given that the set of infected nodes at time *0* is W, can be expressed as*

(6) $$P\left[\mathrm{set}(U(t))=S|\;\mathrm{set}(U(0))=W\right].$$

*The maximum likelihood estimate of set(U(0)) is a solution to*

(7) $$\max_{W\in W_{S}}P\left[\mathrm{set}(U(T))=S|\;\mathrm{set}(U(0))=W\right],$$

*where S⊆V is a given set of infected nodes observed at some independently chosen random time T, and $W_{S}\subseteq 2^{S}$ is a set of hypotheses.*

The single-source detection problem corresponds to the case $W_{S}=\left\{\left\{s\right\}|s\in S\right\}$. In this case, we will show that our proposal (7) can be more meaningful than the existing formulations that optimize

(8) $$\max_{s\in S}P\left[\mathrm{set}\left(U\left(T^{\left(k\right)}\right)\right)=S|\;\mathrm{set}\left(U\left(0\right)\right)=\left\{s\right\}\right],$$

(9) $$\max_{s\in S}\underset{t\geq 0}{\sup}P\left[\mathrm{set}(U(t))=S|\;\mathrm{set}(U(0))=\{s\}\right],$$

where $T^{\left(k\right)}$ is defined in (5). Equation (8) is the ML estimator considered in [14], and Equation (9) is the ML estimator considered in [20].

### 2.2. Fundamental Limitations of Conventional Estimators

We propose our computation of the likelihood probability (6) by summing the probability of all possible infection sequences:

(10) $$\begin{array}{c} P\left[\mathrm{set}(U(t))=S|\;\mathrm{set}(U(0))=W\right]=\sum_{u^{k}\in \Pi_{S|W}}P\left[U\left(t\right)=u^{k}|\;\mathrm{set}\left(U\left(0\right)\right)=W\right], \end{array}$$

where $\Pi_{S|W}$ is the set of permitted infection sequence $u^{k}$ from *W* to *S* satisfying

(11) $$\begin{array}{c} u_{l}\in S\setminus \left(W\cup \mathrm{set}(u^{l-1})\right)\;\mathrm{and}\;\\ \lambda_{u_{l}}\left(W\cup \mathrm{set}\left(u^{l-1}\right)\right)>0,\;\forall l\in \left[k\right]. \end{array}$$

To compute the probability of each permitted infection sequence, define

(12) $$\begin{array}{cc} f_{W,u^{k}}^{\left(l\right)}\left(t\right) & :=P\left[U\left(t\right)\;=\;u_{l}^{k}|\;\mathrm{set}\left(U\left(0\right)\right)\;=\;W\cup \mathrm{set}\left(u^{l-1}\right)\right]. \end{array}$$

$f_{W,u^{k}}^{\left(l\right)}\left(t\right)$ can be computed recursively due to the following recurrence formula:

**Proposition** **1.** *The likelihood probability in (6) can be computed by*

$$\begin{array}{c} P\left[\mathrm{set}(U(t))=S|\;\mathrm{set}(U(0))=W\right]=\sum_{u^{k}\in \Pi_{S|W}}f_{W,u^{k}}^{\left(1\right)}\left(t\right), \end{array}$$

*where*

(13) $$\begin{array}{cc} f_{W,u^{k}}^{\left(l\right)}\left(t\right) & =\left\{\begin{array}{cc} \left(f_{W,u^{k}}^{\left(l+1\right)}*g_{W,u^{l}}\right)\left(t\right) & l\in [k]\\ 1 & l=k+1 \end{array}\right. \end{array}$$

(14) $$\begin{array}{cc} g_{W,u^{l}}\left(t\right) & :=\left.p_{T_{u_{l}}\left(B\right)}\left(t\right)\prod_{v\in \left(V\setminus B\right)\setminus \left\{u_{l}\right\}}P\left[T_{v}\left(B\right)>t\right]\right. \end{array}$$

(15) $$\begin{array}{cc} B & :=W\cup \mathrm{set}\left(u^{l-1}\right). \end{array}$$

**Proof.**  See Appendix A.    □

Through a concrete example, we show that the existing ML estimator (8) and JML estimator (9) considered in [14,20], respectively, both fail to identify the rumor source. In contrast, our proposed estimator (7), which incorporates prior knowledge of the spreading time, can effectively identify the rumor source.

**Example** **1.** *Consider the single-source detection problem in the network in Figure 1, where $\lambda_{01}=\lambda_{12}=1$. Suppose node *1* is the* unknown *rumor source, and at time t, we observe that all other nodes are infected, i.e., S=V={0,1,2}.*

**Proposition** **2.** *For Example 1, we have*

*(i)*  *Both estimators in (8) and (9) are degenerate, since*
$$\begin{array}{cc} \; & \arg\max_{s\in S}P\left[\mathrm{set}\left(U\left(T^{\left(k\right)}\right)\right)=S|\;\mathrm{set}\left(U\left(0\right)\right)=\left\{s\right\}\right]\\ \; & =\arg\max_{s\in S}\underset{t\geq 0}{\sup}P\left[\mathrm{set}(U(t))=S|\;\mathrm{set}(U(0))=\{s\}\right]\\ \; & =S; \end{array}$$
*(ii)*  *For all t∈(0,∞), there is a unique s maximizing*
$$\begin{array}{c} P\left[\mathrm{set}(U(t))=S|\;\mathrm{set}(U(0))=\{s\}\right]; \end{array}$$
*(iii)*  *For all probability distributions on the spreading time T, we have*
$$\begin{array}{cc} \; & \arg\max_{s\in S}P\left[\mathrm{set}(U(T))=S|\;\mathrm{set}(U(0))=\{s\}\right]\\ \; & =\arg\max_{s\in S}P\left[\mathrm{set}(U(t))=S|\;\mathrm{set}(U(0))=\{s\}\right]. \end{array}$$

**Proof.**  See Appendix B.    □

Regarding the maximum likelihood estimator (8) and joint maximum likelihood estimator (9), we observe that they satisfy the following relationship.

**Proposition** **3.** *For S⊆V and s∈S, we have*

$$\begin{array}{cc} \; & \underset{t\geq 0}{\sup}P\left[\mathrm{set}(U(t))=S|\;\mathrm{set}(U(0))=\{s\}\right]\\ \; & =P\left[\mathrm{set}\left(U\left(T^{\left(k\right)}\right)\right)=S|\;\mathrm{set}\left(U\left(0\right)\right)=\left\{s\right\}\right]\underset{t\geq 0}{\sup}\alpha^{\left(s\right)}\left(t,S\right), \end{array}$$

*where*

$$\begin{array}{c} \alpha^{\left(s\right)}\left(t,S\right):=P\left[T^{\left(k\right)}\leq t<T^{\left(k+1\right)}|\;\mathrm{set}\left(U\left(0\right)\right)=\left\{s\right\},\mathrm{set}\left(U\left(T^{\left(k\right)}\right)\right)=S\right]. \end{array}$$

**Proof.**  See Appendix C.    □

**Corollary** **1.** *For S⊆V, we have*

$$\begin{array}{cc} \; & \arg\max_{s\in S}P\left[\mathrm{set}\left(U\left(T^{\left(k\right)}\right)\right)=S|\;\mathrm{set}\left(U\left(0\right)\right)=\left\{s\right\}\right]\\ \; & =\arg\max_{s\in S}\underset{t\geq 0}{\sup}P\left[\mathrm{set}(U(t))=S|\;\mathrm{set}(U(0))=\{s\}\right], \end{array}$$

*if $\alpha^{\left(s\right)}\left(t,S\right)$ is independent of s.*

**Proof.**  See Appendix D.    □

## 3. Problem Formulation

In the following subsections, we give the alternative rate-constrained formulation and the network link model for general both directed and undirected information flow.

### 3.1. Rate-Constrained Model

Consider a communication network on a discrete set *V* of nodes, where ρ(S,A) denotes the multicast capacity from a set S⊆V of source nodes to a set A⊆V of sink nodes. The problem of interest is as follows:

**Definition** **3** (Rate-constrained feasible sources)**.** *Given some rumor with rate r>0 is multicast to a sink A⊆V, the set of inclusionwise minimal feasible sources is defined as*

(16) $$\begin{array}{c} S_{r}\left(A\right):=\mathrm{minimal}\left\{S\subseteq V|\rho \left(S,A\right)\geq r\right\}. \end{array}$$

*for some multicast capacity ρ(S,A) satisfying*

(17) $$\begin{array}{cc} 0 & \overset{\left(a\right)}{\leq }\rho \left(S,A^{\prime }\right)\leq \rho \left(S^{\prime },A\right)\overset{\left(b\right)}{\leq }\infty , \end{array}$$

*for all $S\subseteq S^{\prime }\subseteq V$ and $A\subseteq A^{\prime }\subseteq V$. Equality holds for (a) if S=∅ and for (b) if A=∅.*

This definition identifies the smallest sets of nodes that can collectively support the required rumor spreading rate *r*. Minimality is imposed without loss of generality because ρ(S,A) is non-decreasing in *S*. More explicit formulations in terms of the network links model will be given in Definitions 6 and 7. If the rumor rate *r* is not available, but the size of the source is bounded, one may further consider the following problem instead:

**Definition** **4** (Size-constrained optimal sources)**.** *Given a sink A⊆V, the maximum multicast capacity from a feasible source of size at most k∈[|V|] is defined as*

(18) $$\begin{array}{cc} \rho^{\left(k\right)}\left(A\right) & :=\max\{r>0|S\in S_{r}\left(A\right),|S|\leq k\} \end{array}$$

(19) $$\begin{array}{cc} \; & =\max_{S\subseteq V:|S|\leq k}\rho \left(S,A\right), \end{array}$$

*and $S^{\left(k\right)}\left(A\right)$ is defined as the corresponding set of inclusion-wise minimal solution.*

**Figure 2:**
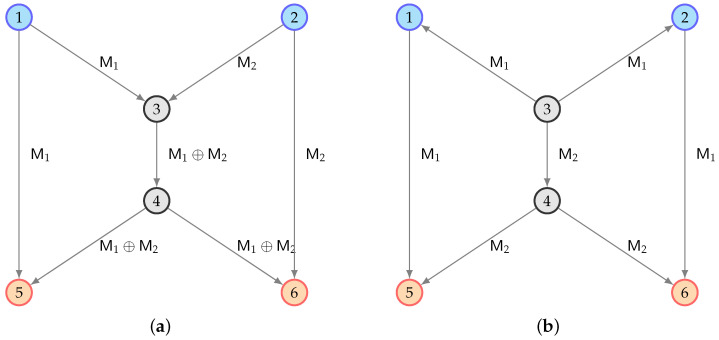
Multicasting two bits $M_{1}$ and $M_{2}$ of rumor from different sources to the sink A:={5,6} over butterfly networks with different link directions. Source nodes are represented by cyan dots, while sinks are in orange. (**a**) Coding for *S* = {1, 2}. (**b**) Routing for *S* = {3}.

This definition is used to determine the highest possible multicast rate under the constraint that the size of the source set does not exceed *k*. The formulation can be illustrated as follows:

**Example** **2** (Directed butterfly network)**.** *Consider the butterfly network in Figure 2a on V:=[6] with independent noiseless channels from node u∈V to w∈V with capacity $\lambda_{uw}=1$ bit for all directed edges (u,w).*

*For A:={5,6}, the usual multi-source multicast rate from $S\in 2^{V}$ can be shown to be*

$$\begin{array}{cc} \rho (S,\{5,6\}) & =\left\{\begin{array}{cc} 0 & S\subseteq \{5,6\}\\ 2 & \{1,2\}\subseteq \;S\\ 1 & \mathrm{otherwise}. \end{array}\right. \end{array}$$

*In particular, the source {1,2} achieves a rate of *2* by network coding as shown in Figure 2a, while other sources in {1,2,3,4} not including {1,2} achieve a rate of *1* by routing. We have*

(20) $$\begin{array}{c} S_{r}\left(\left\{5,6\right\}\right)=\left\{\begin{array}{cc} \left\{\{u\}|1\leq u\leq 4\right\} & r\in (0,1]\\ \left\{\{1,2\}\right\} & r\in (1,2]\\ \emptyset & \mathrm{otherwise}, \end{array}\right. \end{array}$$

(21) $$\begin{array}{c} S^{\left(k\right)}\left(\left\{5,6\right\}\right)=\left\{\begin{array}{cc} \left\{\{u\}|1\leq u\leq 4\right\} & k=1\\ \left\{\{1,2\}\right\} & \mathrm{otherwise}, \end{array}\right. \end{array}$$

*and $\rho^{\left(k\right)}\left(\left\{5,6\right\}\right)=\min\left\{k,2\right\}$. In particular, the source {1,2} can be identified with no ambiguity if the total rate of the rumor exceeds *1*.*

### 3.2. Network Links Model

In Example 2, we illustrated how the multiple multicast rate ρ(S,A) can be computed using a network links model, such as a weighted directed graph. To capture general information flow that goes beyond traditional graphs, we now introduce the following enhanced formulation based on the concept of a polylinking system. This generalization allows us to capture intricate communication structures and multicast capacities, accommodating broader classes of network scenarios beyond simple directed/undirected graphs.

**Definition** **5.** *A directed network links model is a polylinking function $\lambda :{\left(2^{V}\right)}^{2}\mapsto R_{+}$ satisfying [31,32].*

(22) $$\begin{array}{ccc} \begin{array}{cc} \lambda (\emptyset ,B) & =\lambda (B,\emptyset )=0\\ \lambda (B^{\prime },C^{\prime }) & \leq \lambda (B,C) \end{array} & \; & \begin{array}{c} \forall B^{\prime }\subseteq B\subseteq V,\\ C^{\prime }\subseteq C\subseteq V \end{array} \end{array}$$

(23) $$\begin{array}{cccc} \begin{array}{c} \;\sum_{i=1}^{2}\lambda (B_{i},C_{i})\geq \end{array} & \begin{array}{c} \lambda (B_{1}\cap B_{2},C_{1}\cup C_{2})\\ +\lambda (B_{1}\cup B_{2},C_{1}\cap C_{2}) \end{array} & \; & \begin{array}{c} \forall B_{i},C_{i}\subseteq V,\\ i\in [2] \end{array} \end{array}$$

*(22) means λ is normalized and non-decreasing, and (23) is called bisubmodularity. For simplicity, we write $\lambda_{u}\left(B\right):=\lambda \left(B,\left\{u\right\}\right)$.*

This property generalizes the notion of submodularity from single-set functions to functions involving two sets. For submodular functions, adding an element to a larger set yields a smaller marginal gain compared to adding it to a smaller set, a property known as diminishing returns. Bisubmodularity extends this concept to linking functions λ(B,C) by jointly considering the source set *B* and the sink set *C*.

In particular, λ generalizes the following property of graph cuts that gives rise to polynomial-time max-flow min-cut algorithms.

**Proposition** **4.** *f(C):=λ(V∖C,C), called the incut function, is a normalized submodular function, i.e., f(∅)=0, and*

(24) $$\begin{array}{cc} f\left(C_{1}\right)+f\left(C_{2}\right) & \geq f\left(C_{1}\cap C_{2}\right)+f\left(C_{1}\cup C_{2}\right). \end{array}$$

**Proof.**  See Appendix E.    □

The generality of the linking function is illustrated below:

**Example** **3.** *Consider a discrete memoryless deterministic channel $P_{Y_{V}|X_{V}}$, where $X_{u}$ and $Y_{u}$ for u∈V are the discrete channel input and output of u∈V, respectively, and $Y_{V}:=\left(Y_{u}|u\in V\right)$ and $X_{V}:=\left(X_{u}|u\in V\right)$ satisfy*

(25) $$\begin{array}{cc} H(Y_{V}|X_{V}) & =0\;\mathrm{and}\;H\left(X_{V}\right)=\sum_{i\in V}H\left(X_{i}\right). \end{array}$$

*The linking function can be chosen as*

$$\begin{array}{cc} \lambda (B,C) & :=I(X_{B}\wedge Y_{C}|X_{V\setminus B})\;\mathrm{for}\;B,C\subseteq V\\ \; & =H\left(Y_{C}|X_{V\setminus B}\right)=\underset{\left(a\right)}{\underset{︸}{H(Y_{C},X_{V\setminus B})}}-\sum_{u\in V\setminus B}H\left(X_{u}\right). \end{array}$$

*Bisubmodularity in (23) follows immediately from the submodularity of the entropy in (a): (polylinking system is equivalent to based polymatroid [32], i.e., λ(B,C)=f(C|g(B)) for some bijection $g:V\to V^{\prime }$ with $V^{\prime }\cap V=\emptyset$ and a normalized non-decreasing submodular function $f:2^{V\cup V^{\prime }}\to R_{+}$).*

$$\begin{array}{cc} \begin{array}{c} \sum_{i=1}^{2}H(Y_{C_{i}},X_{V\setminus B_{i}})\geq \; \end{array} & \begin{array}{c} H(Y_{C_{1}\cup C_{2}},X_{V\setminus (B_{1}\cap B_{2})})\\ +H(Y_{C_{1}\cap C_{2}},X_{V\setminus (B_{1}\cup B_{2})}) \end{array} \end{array}$$

*The directed network links model is the special case when*

(26) $$\begin{array}{cc} \lambda (B,C) & :=\sum_{u\in B,w\in C}\lambda_{uw}, \end{array}$$

*where $\lambda_{uw}$ is the capacity of an independent noiseless channel from u∈V to w∈V.*

We consider the rumor to be a vector of independent messages, with one from a different source node. The multicast rate ρ can be efficiently computed from λ using the max-flow min-cut results (see for instance [21]), which were extended to the cyclic linking network model in [33], as follows:

**Definition** **6.** *For directed networks, the maximum multi-source multicast rate is*

(27) $$\begin{array}{c} \begin{array}{cc} \rho_{\mathrm{ms}}\left(S,A\right) & :=\max\{r\left(S\right)|r_{V}\in R_{+}^{V},\\ \; & \;r(B)\leq \lambda (B,V\setminus B)\;\forall B\subseteq V:B⊉A\}, \end{array} \end{array}$$

*where $r_{V}:=\left(r_{i}|i\in V\right)$, and $r\left(B\right):=\sum_{u\in B}r_{u}$.*

*If source nodes can collude to encode the rumor, the capacity becomes the maximum single-source multicast rate*

(28) $$\begin{array}{cc} \rho_{\mathrm{ss}}\left(S,A\right) & :=\min_{B\subseteq V:S\subseteq B⊉A}\lambda \left(B,V\setminus B\right). \end{array}$$

*If the sink nodes can collude to recover the rumor, the capacity becomes the maximum multiple access rate*

(29) $$\begin{array}{c} \begin{array}{cc} \rho_{\mathrm{ma}}\left(S,A\right) & :=\max\{r\left(S\right)|r_{V}\in R_{+}^{V},\\ \; & \;r(B)\leq \lambda (B,V\setminus B)\;\forall B\subseteq V\setminus A\}. \end{array} \end{array}$$

*If the source nodes and sink nodes can collude respectively, the capacity becomes the maximum unicast rate*

(30) $$\begin{array}{cc} \rho_{\mathrm{uc}}\left(S,A\right) & :=\min_{B\subseteq V\setminus A:S\subseteq B}\lambda \left(B,V\setminus B\right). \end{array}$$

For undirected networks, the maximum multicast rates can be computed using the result of the matroid undirected network in [34]. More precisely, the directed network rates can be modified by an additional optimization over choice of the direction, which can be modeled as a choice of the base $x_{V}\in B\left(h\right)$ of a normalized non-decreasing submodular function $h:2^{V}\mapsto R_{+}$ with the cut function replaced as follows

$$\begin{array}{c} \lambda (B,V\setminus B)=h(V\setminus B)-x(V\setminus B). \end{array}$$

**Definition** **7** (Undirected communication capacities)**.** *The capacities in Definition 6 for undirected networks are*

(31) $$\begin{array}{c} \begin{array}{cc} {\tilde{\rho }}_{\mathrm{ms}}\left(S,A\right) & :=\max\{r\left(S\right)|r_{V}\in R_{+}^{V},\\ \; & \;r(B)\leq \lambda (B,V\setminus B)\;\forall B\subseteq V:B⊉A\} \end{array} \end{array}$$

(32) $$\begin{array}{cc} {\tilde{\rho }}_{\mathrm{ss}}\left(S,A\right) & :=\min_{B\subseteq V:S\subseteq B⊉A}\lambda \left(B,V\setminus B\right). \end{array}$$

(33) $$\begin{array}{c} \begin{array}{cc} {\tilde{\rho }}_{\mathrm{ma}}\left(S,A\right) & :=\max\{r\left(S\right)|r_{V}\in R_{+}^{V},\\ \; & \;r(B)\leq \lambda (B,V\setminus B)\;\forall B\subseteq V\setminus A\}. \end{array} \end{array}$$

(34) $$\begin{array}{cc} {\tilde{\rho }}_{\mathrm{uc}}\left(S,A\right) & :=\min_{B\subseteq V\setminus A:S\subseteq B}\lambda \left(B,V\setminus B\right). \end{array}$$

The source detection problem for information flow is very rich, as one may consider different possibilities of collusion in the calculations of the capacities. Furthermore, the different capacities are polynomial-time computable and can be viewed as bounds on each other as follows:

**Proposition** **5.** *The multicast rates are related as follows:*

(35) $$\begin{array}{cc} \rho_{\mathrm{ss}}\left(S,A\right),\rho_{\mathrm{ma}}\left(S,A\right) & \in [\rho_{\mathrm{ms}}\left(S,A\right),\rho_{\mathrm{uc}}\left(S,A\right)], \end{array}$$

(36) $$\begin{array}{cc} {\tilde{\rho }}_{\mathrm{ss}}\left(S,A\right),{\tilde{\rho }}_{\mathrm{ma}}\left(S,A\right) & \in [{\tilde{\rho }}_{\mathrm{ms}}\left(S,A\right),{\tilde{\rho }}_{\mathrm{uc}}\left(S,A\right)]. \end{array}$$

*Furthermore, these rates are computable in polynomial time.*

**Proof.**  See Appendix F.    □

The rate-constrained source detection problem for undirected networks is illustrated below:

**Example** **4** (Undirected butterfly network)**.** *Consider an undirected butterfly network where each link can be redirected arbitrarily and fractionally. Figure 2 shows two different ways to direct the network. For A:={5,6}, the multi-source multicast rate from $S\in 2^{V}$ is*

(37) $$\begin{array}{cc} \rho (S,\{5,6\}) & =\left\{\begin{array}{cc} 0 & S=\emptyset ,\\ 1 & S\in \{\{1\},\{2\}\},\\ 2 & \mathrm{otherwise}. \end{array}\right. \end{array}$$

*In addition to the coding solution for the source {1,2} shown in Figure 2a, Figure 2b shows how a rate of *2* can be achieved by routing from source {3}. It follows that*

(38) $$\begin{array}{c} S_{r}\left(\left\{5,6\right\}\right)=\left\{\begin{array}{cc} \left\{\{u\}|1\leq u\leq 6\right\} & r\in (0,1]\\ \left\{\{1,2\},\{u\}|3\leq u\leq 6\right\} & r\in (1,2]\\ \emptyset & \mathrm{otherwise}. \end{array}\right. \end{array}$$

*$\rho^{\left(k\right)}\left(\left\{5,6\right\}\right)=2$ for k∈[6] with $S^{\left(1\right)}\left(\left\{5,6\right\}\right)=\left\{\left\{u\right\}|3\leq u\leq 6\right\}$ and $S^{\left(k\right)}\left(\left\{5,6\right\}\right)=\left\{\left\{1,2\right\},\left\{u\right\}|3\leq u\leq 6\right\},2\leq k\leq 6$. In particular, when the rumor rate exceeds *1*, the set {1,2} is not the only possible source, and there are simpler feasible sources such as {3}.*

## 4. Main Results

### 4.1. Rate-Constrained Source Selection Algorithms

We first consider a backward elimination algorithm to find a feasible source in $S_{r}\left(A\right)$ in (16) for the rate-constrained model.

**Proposition** **6.** *The algorithm in Listing 1 gives a feasible solution in $S_{r}\left(A\right)$, if any, in time polynomial in |V|.*

**Proof.**  Correctness follows from the fact that ρ(S,A) is non-decreasing in *S* (17). Since ρ(S,A) is computable in polynomial time by Proposition 5, and there are at most |V| such computations, the overall complexity is still polynomial.    □

**Listing 1.** Backward elimination algorithm.   1 def feasible_source (*A*):   2     if *ρ*(*V*,*A*) < *r*: return None   3     *S* = *V*   4     for *u* in *V*:   5         if *ρ*(*S*\{*u*}, *A*) ≥ *r*: *S* = *S*\{*u*}   6      return *S*

When the rate *r* is not available, but the size of the source set is constrained by *k*, a forward search algorithm similar to the one for submodular function maximization can be used to approximate $\rho^{\left(k\right)}\left(A\right)$ in (4).

The above greedy forward search algorithm has been shown to be a good approximation for the multi-source unicast scenario since $\rho_{uc}\left(S,A\right)$ is submodular in *S*, and $\rho^{\left(k\right)}\left(S,A\right)$ becomes a size-constrained submodular function maximization.

**Proposition** **7.** *The algorithm in Listing 2 gives a lower bound of $\rho^{\left(k\right)}$ in time polynomial in |V|. Furthermore, if ρ(S,A) is submodular in S, the lower bound is at least $\left(1-1/e\right)\rho^{\left(k\right)}\left(S,A\right)$.*

**Proof.**  Since ρ(S,A) is computable in polynomial time by Proposition 5, and there are at most ${\left|V\right|}^{2}$ such computations, the overall complexity is still polynomial. If $\rho^{\left(k\right)}\left(S,A\right)$ is submodular, the problem becomes the size-constrained submodular function maximization. The approximation factor was given by [35].    □

**Listing 2.** Forward search algorithm.  1 def approximate_rho (k):  2     *S*, *r*, *u* = ∅, 0, None  3     while |*S*| < *k*:  4         for *w* in *V*\*S*:  5             if *ρ*(*S*∪{*w*}, *A*) ≥ *r*:  6                 *u*, *r* = *w*, *ρ*(*S*∪{*w*}, *A*)  7             *S* = *S*∪{*u*}  8     return *r*

### 4.2. Source Feasibility Under Multicast Rate Constraints

To simplify the calculation of (31), we use the max-flow min-cut result from network coding [21] and apply the node contraction to find the minimum cut value λ(B,V∖B) from source nodes to sink nodes.

(39) $$\begin{array}{cc} \min_{B\subseteq V}\lambda \left(B,V\setminus B\right) & =\min_{j\in A}\min_{B\subseteq V\setminus \{j\}}\lambda \left(B,\left\{j\right\}\right) \end{array}$$

(40) $$\begin{array}{cc} \; & =\min_{j\in A}\mathrm{mincut}\left(s,j\right), \end{array}$$

where *s* is the node by node contraction in *B*. Note that the simplification can be used in all the cases by adding the corresponding constraints.

Then, we reproduced (The source code of our experiments can be found at https://github.com/ZimengInfo/InfodemicSource, accessed on 11 August 2025) the result from Example 2 using (31) and the backward elimination in Listing 1. In this setup, the sink set was fixed to A={5,6}. By enumerating the multicast rate values, the feasible sources were the minimal set achieving the target rate. This exactly matches the optimal solution given in Example 2, thereby verifying the correctness of our implementation and the soundness of the backward elimination approach. For further verification, (31) is also treated as a linear programming problem with submodular constraints to show the precise solutions.

We extend our feasibility experiment beyond the butterfly network to synthetic graphs commonly used in network modeling: Barabási–Albert (BA) [36], Watts–Strogatz (WS) [37], and Erdős–Rényi (ER) [38,39], each with 20 nodes. These graphs were first generated as undirected, cleaned for weak connectivity, and then converted to directed graphs with consistent flow direction (from lower- to higher-indexed nodes). Unit capacities were assigned to all edges. For each graph, we selected a sink set *A* appropriate to its structure. Specifically, for the BA graph, which has hubs forming early in the index sequence, we selected the first two nodes, i.e., {0,1}, as sinks. For the other two, which have more uniform edge direction distributions, we selected the last two nodes, i.e., {18,19}. This ensures that the sinks are actually reachable from potential source nodes.

We then applied the backward elimination algorithm using the contraction-based multicast rate to test for feasible source sets under target rate thresholds r∈{0.5,1.0,2.0,3.0,6.0}. The goal was to find a minimal source set *S* such that $\rho_{\mathrm{ms}}\left(S,A\right)\geq r$. From Table 1, we observe that all graphs admit small feasible sets for low target rates. As the rate increases, the feasible sets grow in size and, for the ER graph, become infeasible at higher thresholds. The BA graph, benefiting from its scale-free structure, typically requires fewer sources to reach the sinks than WS and ER graphs. These results demonstrate the practical behavior of our source selection algorithm and confirm the influence of graph topology on multicast feasibility.

### 4.3. Computational and Structural Behavior of Lazy–Greedy Forward Search

In the greedy forward search, the objective is to find a set *S* of source nodes that maximizes the multicast rate ρ(S,A), where *A* is the sink set. At each step, we select the node *w* that provides the maximum marginal gain to the current set *S*, where the marginal gain $\Delta_{w}\left(S\right)$ is defined as [40]:

(41) $$\Delta_{w}\left(S\right)=\rho \left(S\cup \left\{w\right\},A\right)-\rho \left(S,A\right).$$

The marginal gain represents the additional benefit gained by adding node *w* to the set *S*. For each iteration, the greedy algorithm evaluates all nodes in V∖S and selects the one with the highest marginal gain. This process continues until the desired number of source nodes *k* are selected, see Listing 2.

We also propose the lazy–greedy forward search algorithm, which reduces the number of redundant marginal gain evaluations by caching and reusing previously computed values. The core idea is that once a node *w* has been identified as the best candidate in one iteration, it does not need to be re-evaluated unless it rises to the top of the priority queue again due to changes in the current source set *S*.

The lazy–greedy algorithm uses a priority queue to store nodes along with their cached marginal gains. At each step, the node with the largest cached marginal gain is selected, and the marginal gain is recomputed only if the cached value is outdated. This reduces the number of recalculations, especially for nodes that are not frequently selected, see Listing 3.
**Listing 3.** Lazy–greedy forward search algorithm.   1  def approximate_rho(k):   2      *S*, *r* = ∅, 0   3      PQ = [(− *ρ*({*W*}, *A*), *w*) for *w* in *V*]   4      heapify(PQ)   5      while |*S*| < *k* and PQ ≠ ∅:   6          *g*, *w* = heappop(PQ)   7          if *w* ∈ *S*: continue   8          Δ = *ρ*(*S*∪{*w*}, *A*)− *r*   9          if Δ < −*g*:  10              heappush(PQ, (−Δ, *w*))  11              continue  12          S = *S*∪{*w*}  13          *r* = *r* + Δ  14      return r
where PQ is the priority queue, Δ is the marginal gain, and *g* is the cached gain. The lazy–greedy approach introduces several improvements over the original greedy algorithm:

- Efficiency: The primary advantage of the lazy–greedy algorithm is its efficiency in terms of the computational cost. By caching the marginal gains and only recomputing them when necessary, the lazy–greedy algorithm reduces the number of oracle calls and heap operations compared to the original greedy approach. While the greedy algorithm evaluates every node in V∖S in each iteration, the lazy–greedy algorithm evaluates only those nodes that are most likely to provide the largest marginal gain, reducing unnecessary computations.
- Time Complexity: The original greedy algorithm has a time complexity of O(k·|V|) because it must evaluate all nodes in V∖S during each iteration. In contrast, the lazy–greedy version has $O(k\cdot \log|V|+\sum_{w}\tau \left(w\right)\log|V|)$, where τ is the number of times node *w* is evaluated.
- Approximation Guarantee: Both the greedy and lazy–greedy algorithms maintain the same approximation factor of 1−1/e when ρ is submodular, as demonstrated by Proposition 5.

Now let us evaluate the practical scalability and rate behavior with Listing 3, which we use throughout this work to approximate the maximum multicast rate $\rho (S^{\left(k\right)}A,A)$ under a source budget constraint. This algorithm is designed to exploit the diminishing returns property of monotonic rate functions via a priority queue and marginal gain caching mechanism.

**Figure 3:**
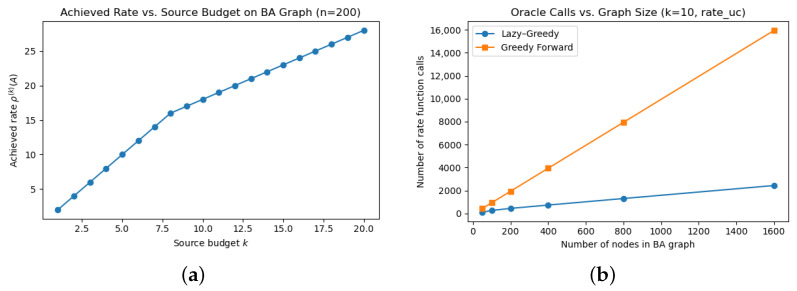
(**a**) Achieved multicast rate $\rho (S^{\left(k\right)}A,A)$ using lazy–greedy forward search on a 200-node Barabási–Albert graph. (**b**) Number of oracle calls made by lazy–greedy and plain greedy forward search algorithms as a function of graph size *n*, with fixed source budget k=10, using the unicast rate model (rate_uc).

In the first experiment, we explore how the achieved multicast rate $\rho (S^{\left(k\right)}A,A)$ evolves with increasing source budget *k*. We generate the Barabási–Albert graphs, which is a good representation of social networks [36], fix the graph size to n=200, set a fixed sink set A={0,1}, and run the lazy–greedy algorithm for k∈{1,2,…,20}. The results, shown in Figure 3a, indicate a steady increase in the achieved rate up to approximately 11.0, after which the curve saturates. This saturation point reflects the inherent capacity limit (min-cut) between the selected source set and the sinks. The experiment confirms that the algorithm prioritizes high-impact nodes early and naturally exposes network bottlenecks, making it well-suited for capacity-aware source detection, i.e., the size-constrained optimal source problem.

We then assess how the number of oracle calls scales with the graph size. We generate BA graphs of increasing size n∈{50,100,200,400,800,1600}, with unit edge capacities and maintain the sink set A={1,0}. For each graph, we fix the source budget k=10 and count the number of calls to $\rho_{\mathrm{uc}}$ during the execution of both the lazy–greedy and plain greedy algorithms. As shown in Figure 3b, both methods exhibit linear growth in the number of oracle calls as the number of nodes increases. This is consistent with the theoretical bounds, since the number of calls is O(k·|V|), and *k* is held constant. Importantly, the lazy–greedy algorithm consistency performs fewer oracle calls due to its ability to reuse cached marginal gains, confirming its practical scalability benefits.

These experiments collectively demonstrate that the lazy–greedy forward search algorithm is both computationally efficient and behaviorally robust. It scales linearly with the graph size under fixed budget constraints and achieves near-optimal rates without exhaustive evaluation. These properties make it a practical choice for source selection tasks in large-scale networks.

## 5. Discussion

This study revisits the problem of source detection and underscores the significant limitations of the conventional likelihood-based estimators. Theoretical analysis reveals that both maximum likelihood (ML) and joint maximum likelihood (JML) estimators exhibit degeneracy even in straightforward simple topologies, such as a three-node line graph. To address this issue, we have reformulated the source detection problem through the framework of rate-constrained multicast capacity, drawing upon network flow theory and polylinking systems. By employing multicast rate functions ρ(S,A), we are able to precisely identify the feasible sources in a more general graphical structure.

The experimental results substantiate both the theoretical correctness and practical applicability of this framework. The backward elimination algorithm effectively identifies minimal feasible sources within a specified rate threshold, as shown in the example of the butterfly network. On the computational front, the lazy–greedy forward search algorithm achieves near-optimal rate performance while adhering to cardinality constraints. Notably, our empirical analysis indicates that the number of oracle calls increases linearly with the size of the graph when the source budget *k* remains fixed, thereby confirming the scalability of the proposed approach. Furthermore, the rate attained through greedy source selection quickly approaches saturation as *k* increases, aligning with the network’s min-cut bound and demonstrating the model’s capacity to reveal fundamental flow bottlenecks.

Our framework assumes access to either the rumor spreading rate *r* or the size of the source set *k*. If neither is available, the method cannot be directly applied. However, in practical cases, such as epidemic tracking, the set of infected nodes is observed incrementally, rather than all at once. As new infected nodes are discovered, the underlying graph and infection snapshot can be updated, allowing the source detection algorithms to be applied recursively. Thus, developing a systematic version of this recursive strategy presents a promising direction for future work.

Another avenue for future research may involve applying this rate-constrained model to large-scale real-world networks. This would involve challenges such as determining optimal guarantee for the submodular function maximization with Listing 2 in the most general case of $\rho_{ms}\left(S,A\right)$, as well as exploring the GPU-accelerated max-flow min-cut algorithm to improve the computational efficiency.

## 6. Conclusions

We introduced a rate-constrained framework for infodemic source detection, motivated by the failure of maximum likelihood-based estimators in even simple networks. Our approach leverages the structure of multicast capacity and information flow to define source feasibility and optimize source selection. We proposed polynomial-time algorithms based on backward elimination and forward greedy approximation.

Our results show that the proposed methods are effective and scalable, achieving near-optimal rates and revealing structural bottlenecks in both theoretical examples and synthetic graphs. The modeling flexibility and algorithmic efficiency of our framework provide a principled foundation for future extensions.

## Figures and Tables

**Table 1:** Feasible source sets found by backward elimination on synthetic graphs using the contraction-based rate function $\rho_{\mathrm{ms}}$. Each row shows whether a feasible source set exists for a given rate threshold, along with the size and node indices of the selected source set.

Graph	Target Rate	Feasible	Set Size	Source Set
BA	0.5	Yes	1	{19}
BA	1.0	Yes	1	{19}
BA	2.0	Yes	1	{18}
BA	3.0	Yes	3	{17, 18, 19}
BA	6.0	Yes	5	{11, 12, 15, 17, 18}
WS	0.5	Yes	1	{17}
WS	1.0	Yes	1	{17}
WS	2.0	Yes	2	{11, 16}
WS	3.0	Yes	3	{10, 11, 13}
WS	6.0	No	–	–
ER	0.5	Yes	1	{17}
ER	1.0	Yes	1	{17}
ER	2.0	Yes	1	{17}
ER	3.0	Yes	3	{16, 18, 19}
ER	6.0	No	–	–

## Data Availability

The original contributions presented in this study are included in the article. Further inquiries can be directed to the corresponding authors.

## Appendix A. Proof of Proposition 1

**Proof.** We first contract all nodes in *W* into a single node $u_{0}$ without loss of generality so that the infection process can be analyzed from a single source. Consider any infection sequence $u^{k}\in \Pi_{S|W}$. For such a sequence, we expand the probability of observing $U\left(t\right)=u_{l}^{k}$ step by step. Using the chain rule, we obtain

$$\begin{array}{cc} \; & \;P\left[U\left(t\right)=u_{l}^{k}|\mathrm{set}\left(U\left(0\right)\right)=\mathrm{set}\left(u_{0}^{l-1}\right)\right]\\ \; & =\int_{\tau =0}^{t}P\left[U\left(t\right)=u_{l}^{k}|U\left(0\right)=u_{0}^{l-1},T_{u_{l}}\left(\mathrm{set}\left(u_{0}^{l-1}\right)\right)=\tau ,\right.\\ & \;\left.T_{v}\left(\mathrm{set}\left(u_{0}^{l-1}\right)\right)>\tau ,v\in V\setminus \mathrm{set}\left(u_{0}^{l}\right)\right]\\ \; & \;\;\;\times P\left[T_{v}\left(\mathrm{set}\left(u_{0}^{l-1}\right)\right)>\tau ,v\in V\setminus \mathrm{set}\left(u_{0}^{l}\right)\right]dP_{T_{u_{l}}\left(\mathrm{set}\left(u_{0}^{l-1}\right)\right)}\left(\tau \right). \end{array}$$

Here, we condition on the event that node $u_{l}$ becomes infected at time τ while all other nodes remain uninfected until τ. By the memoryless property of exponential random variables, the process after time τ is equivalent to a fresh infection process starting from $u_{l}$. Thus, the above equals

$$\begin{array}{cc} \; & \int_{\tau =0}^{t}P\left[U\left(t-\tau \right)=u_{l+1}^{k}|U\left(0\right)=u_{0}^{l}\right]\\ \; & \;\;\;\times P\left[T_{v}\left(\mathrm{set}\left(u_{0}^{l-1}\right)\right)>\tau ,v\in V\setminus \mathrm{set}\left(u_{0}^{l}\right)\right]dP_{T_{u_{l}}\left(\mathrm{set}\left(u_{0}^{l-1}\right)\right)}\left(\tau \right). \end{array}$$

Because infection times are mutually independent across nodes, the second term factorizes into a product, leading to

$$\begin{array}{cc} \; & \int_{\tau =0}^{t}P\left[U\left(t-\tau \right)=u_{l+1}^{k}|U\left(0\right)=u_{0}^{l}\right]\\ \; & \;\;\;\times \prod_{v\in \left(V\setminus B\right)\setminus \left\{u_{l}\right\}}P\left[T_{v}\left(B\right)>\tau \right]p_{T_{u_{l}}\left(B\right)}\left(\tau \right), \end{array}$$

where $B:=W\cup \mathrm{set}(u^{l-1})$. This expression exactly matches the convolution form

$$\begin{array}{c} f_{W,u^{k}}^{\left(1\right)}=\left(f_{W,u^{k}}^{\left(l+1\right)}*g_{W,u^{l}}\right)\left(t\right), \end{array}$$

where $g_{W,u^{l}}\left(t\right):=p_{T_{u_{l}}\left(B\right)}\left(t\right)\prod_{v\in \left(V\setminus B\right)\setminus \left\{u_{l}\right\}}P\left[T_{v}\left(B\right)>t\right]$. For the base case l=k+1, all nodes in *S* are infected, so the recursion terminates with

$$\begin{array}{c} f_{W,u^{k}}^{\left(k+1\right)}\left(t\right)=1. \end{array}$$

This proves the desired recurrence relation. □

## Appendix B. Proof of Proposition 2

**Proof.** For the ML estimator (8), we have from (6) that

$$\begin{array}{cc} \; & P\left[\mathrm{set}\left(U\left(T^{\left(3\right)}\right)\right)=\left\{0,1,2\right\}|\mathrm{set}\left(U\left(0\right)\right)=\left\{0\right\}\right]\\ \; & =1, \end{array}$$

since (0,1,2) is the only possible spreading sequence of size 3 if we assume node 0 is the rumor source.

$$\begin{array}{cc} \; & P\left[\mathrm{set}\left(U\left(T^{\left(3\right)}\right)\right)=\left\{0,1,2\right\}|\mathrm{set}\left(U\left(0\right)\right)=\left\{1\right\}\right]\\ \; & =1, \end{array}$$

since {0,1,2} is the only possible set of size 3 in the network assuming node 1 is the rumor source. By symmetry, we have

$$\begin{array}{cc} \; & P\left[\mathrm{set}\left(U\left(T^{\left(3\right)}\right)\right)=\left\{0,1,2\right\}|\mathrm{set}\left(U\left(0\right)\right)=\left\{2\right\}\right]\\ \; & =P\left[\mathrm{set}\left(U\left(T^{\left(3\right)}\right)\right)=\left\{0,1,2\right\}|\mathrm{set}\left(U\left(0\right)\right)=\left\{0\right\}\right]\\ \; & =1. \end{array}$$

It then follows that each node in {0,1,2} has the same probability of being the rumor source, i.e., $\arg\max_{s\in S}P\left[\mathrm{set}\left(U\left(T^{\left(k\right)}\right)\right)=S|\mathrm{set}\left(U\left(0\right)\right)=\left\{s\right\}\right]=S$. For the JML estimator (9), we have from (10) that

$$\begin{array}{cc} \; & P\left[\mathrm{set}\left(U\left(t\right)\right)=S|\mathrm{set}\left(U\left(0\right)\right)=\left\{0\right\}\right]\\ \; & =\sum_{u^{2}\in \Pi_{\left\{0,1,2\}|\{0\right\}}}P\left[U\left(t\right)=u^{2}|\mathrm{set}\left(U\left(0\right)\right)=\left\{0\right\}\right]\\ \; & =P\left[U(t)=(1,2)|\mathrm{set}(U(0))=\{0\}\right]\\ \; & =\left(1*e^{-t}\right)*e^{-t}\\ \; & =\int_{\tau =0}^{t}\left[1-e^{-(t-\tau )}\right]e^{-\tau }d\tau \\ \; & ={\left.-e^{-\tau }-e^{-t}\tau \right|}_{\tau =0}^{t}\\ \; & =-te^{-t}-e^{-t}+1. \end{array}$$

$$\begin{array}{cc} \; & P\left[\mathrm{set}\left(U\left(t\right)\right)=S|\mathrm{set}\left(U\left(0\right)\right)=\left\{1\right\}\right]\\ \; & =\sum_{u^{2}\in \Pi_{\left\{0,1,2\}|\{1\right\}}}P\left[U\left(t\right)=u^{2}|\mathrm{set}\left(U\left(0\right)\right)=\left\{1\right\}\right]\\ \; & =P\left[U(t)=(0,2)|\mathrm{set}(U(0))=\{1\}\right]\\ \; & \;+P\left[U(t)=(2,0)|\mathrm{set}(U(0))=\{1\}\right]\\ \; & =\left(1*e^{-t}\right)*\left(e^{-t}\times e^{-t}\right)+\left(1*e^{-t}\right)*\left(e^{-t}\times e^{-t}\right)\\ \; & =2\int_{\tau =0}^{t}\left[1-e^{-(t-\tau )}\right]e^{-2\tau }d\tau \\ \; & ={\left.-e^{-2\tau }+2e^{-t-\tau }\right|}_{\tau =0}^{t}\\ \; & =e^{-2t}-2e^{-t}+1. \end{array}$$

By symmetry, we have

$$\begin{array}{cc} \; & P\left[\mathrm{set}\left(U\left(t\right)\right)=S|\mathrm{set}\left(U\left(0\right)\right)=\left\{2\right\}\right]\\ \; & =P\left[\mathrm{set}\left(U\left(t\right)\right)=S|\mathrm{set}\left(U\left(0\right)\right)=\left\{0\right\}\right]\\ \; & =-te^{-t}-e^{-t}+1. \end{array}$$

Altogether, the supremums over t≥0 of the above three estimators are all 1. Hence, each node in {0,1,2} has the equal probability to be the rumor source, i.e.,

$$\arg\max_{s\in S}\underset{t\geq 0}{\sup}P\left[\mathrm{set}\left(U\left(t\right)\right)=S|\mathrm{set}\left(U\left(0\right)\right)=\left\{s\right\}\right]=S.$$

It follows from the above that

$$\begin{array}{cc} \; & \;P\left[\mathrm{set}\left(U\left(t\right)\right)=S|\mathrm{set}\left(U\left(0\right)\right)=\left\{1\right\}\right]\\ \; & \;-P\left[\mathrm{set}\left(U\left(t\right)\right)=S|\mathrm{set}\left(U\left(0\right)\right)=\left\{0\right\}\right]\\ \; & =e^{-2t}-e^{-t}+te^{-t}\\ \; & =e^{-t}\left[t-(1-e^{-t})\right]. \end{array}$$

It can be easily verified that $t\geq 1-e^{-t}$. Thus, we obtain

$$\begin{array}{cc} \; & P\left[\mathrm{set}\left(U\left(t\right)\right)=S|\mathrm{set}\left(U\left(0\right)\right)=\left\{1\right\}\right]\\ \; & >P\left[\mathrm{set}\left(U\left(t\right)\right)=S|\mathrm{set}\left(U\left(0\right)\right)=\left\{0\right\}\right]\\ \; & =P\left[\mathrm{set}\left(U\left(t\right)\right)=S|\mathrm{set}\left(U\left(0\right)\right)=\left\{2\right\}\right],\;\forall t>0, \end{array}$$

which implies node 1 is the unique *s* maximizing $P\left[\mathrm{set}\left(U\left(t\right)\right)=S|\mathrm{set}\left(U\left(0\right)\right)=\left\{s\right\}\right]$ for all t∈(0,∞). Furthermore, for any probability distribution on the spreading time, the above inequality implies

$$\begin{array}{cc} \; & P\left[\mathrm{set}\left(U\left(T\right)\right)=S|\mathrm{set}\left(U\left(0\right)\right)=\left\{1\right\}\right]\\ \; & >\left[\mathrm{set}\left(U\left(T\right)\right)=S|\mathrm{set}\left(U\left(0\right)\right)=\left\{0\right\}\right]\\ \; & =\left[\mathrm{set}\left(U\left(T\right)\right)=S|\mathrm{set}\left(U\left(0\right)\right)=\left\{2\right\}\right]. \end{array}$$

Thus, we have

$$\begin{array}{cc} \; & \arg\max_{s\in S}P\left[\mathrm{set}\left(U\left(T\right)\right)=S|\mathrm{set}\left(U\left(0\right)\right)=\left\{s\right\}\right]\\ \; & =\arg\max_{s\in S}P\left[\mathrm{set}\left(U\left(t\right)\right)=S|\mathrm{set}\left(U\left(0\right)\right)=\left\{s\right\}\right]\\ \; & =\{1\}, \end{array}$$

for all probability distributions on the spreading time. □

## Appendix C. Proof of Proposition 3

**Proof.** We begin with the definition of the likelihood probability in (6):

$$\begin{array}{c} P\left[\mathrm{set}(U(t))=S|\;\mathrm{set}(U(0))=\{s\}\right]. \end{array}$$

Step 1: Decomposition using event equivalence. The event set(U(t))=S is equivalent to the joint occurrence of the event in which the first *k* infected nodes from the set *S* and the observation time *t* fall between the infection times of the *k*-th and (k+1)-th nodes. Therefore, we can rewrite the probability as
  $$\begin{array}{cc} \; & P\left[\mathrm{set}\left(U\left(T^{\left(k\right)}\right)\right)=S,T^{\left(k\right)}\leq t,T^{\left(k+1\right)}>t|\;\mathrm{set}\left(U\left(0\right)\right)=\left\{s\right\}\right]. \end{array}$$
Step 2: Chain rule factorization. Applying the chain rule, we separate the probability into two terms:
  $$\begin{array}{cc} \; & P\left[\mathrm{set}\left(U\left(T^{\left(k\right)}\right)\right)=S|\;\mathrm{set}\left(U\left(0\right)\right)=\left\{s\right\}\right]\\ \; & \times P\left[T^{\left(k\right)}\leq t<T^{\left(k+1\right)}|\;\mathrm{set}\left(U\left(0\right)\right)=\left\{s\right\},\mathrm{set}\left(U\left(T^{\left(k\right)}\right)\right)=S\right]. \end{array}$$
  Here, the first term depends only on the infection sequence up to size *k*, while the second term captures the conditional timing information.
Step 3: Taking the supremum over *t*. We now take the supremum over all observation times t≥0. Since the first term does not depend on *t*, the supremum only affects the second term:
  $$\begin{array}{cc} \; & \underset{t\geq 0}{\sup}P\left[\mathrm{set}(U(t))=S|\mathrm{set}(U(0))=\{s\}\right]\\ \; & =P\left[\mathrm{set}\left(U\left(T^{\left(k\right)}\right)\right)=S|\;\mathrm{set}\left(U\left(0\right)\right)=\left\{s\right\}\right]\times \underset{t\geq 0}{\sup}\alpha \left(s\right)\left(t,s\right), \end{array}$$
  where
  $$\begin{array}{c} \alpha \left(s\right)\left(t,S\right):=P\left[T^{\left(k\right)}\leq t<T^{\left(k+1\right)}|\;\mathrm{set}\left(U\left(0\right)\right)=\left\{s\right\},\mathrm{set}\left(U\left(T^{\left(k\right)}\right)\right)=S\right]. \end{array}$$
  This shows that the supremum of the JML estimator factorizes into the ML term and a timing factor α(s)(t,S). □

## Appendix D. Proof of Corollary 1

**Proof.** We wish to compare the two argmax expressions in (8) and (9). Starting by recalling Appendix C, which rewrites the joint maximum likelihood (JML) (9) objective as the product of two factors:

$$\begin{array}{cc} \; & \underset{t\geq 0}{\sup}P\left[\mathrm{set}(U(t))=S|\mathrm{set}(U(0))=\{s\}\right]\\ \; & =P\left[\mathrm{set}\left(U\left(T^{\left(k\right)}\right)\right)=S|\;\mathrm{set}\left(U\left(0\right)\right)=\left\{s\right\}\right]\times \underset{t\geq 0}{\sup}\alpha \left(s\right)\left(t,s\right), \end{array}$$

where

$$\begin{array}{c} \alpha \left(s\right)\left(t,S\right):=P\left[T^{\left(k\right)}\leq t<T^{\left(k+1\right)}|\;\mathrm{set}\left(U\left(0\right)\right)=\left\{s\right\},\mathrm{set}\left(U\left(T^{\left(k\right)}\right)\right)=S\right], \end{array}$$

and the first term is exactly the likelihood appearing in (8). When $\alpha^{\left(s\right)}\left(t,S\right)$ is independent of the candidate source node *s*, the supremum $\sup_{t\geq 0}\alpha^{\left(s\right)}\left(t,S\right)$ is a constant with respect to *s* and hence cancels out when taking the argmax over s∈S. □

## Appendix E. Proof of Proposition 4

**Proof.** We firstly prove the normalization. By the definition of *f* and (22),

f(∅)=λ(V∖∅,∅)=λ(V,∅)=0.

We then continue with the proof of submodularity. Since

$$\begin{array}{cc} \; & V\setminus \left(C_{1}\cap C_{2}\right)=\left(V\setminus C_{1}\right)\cup \left(V\setminus C_{2}\right),\\ \; & V\setminus \left(C_{1}\cup C_{2}\right)=\left(V\setminus C_{1}\right)\cap \left(V\setminus C_{2}\right), \end{array}$$

the inequality can be reformulated by

$$\begin{array}{cc} \; & \lambda \left(V\setminus C_{1},C_{1}\right)+\lambda \left(V\setminus C_{2},C_{2}\right)\\ \; & \geq \lambda \left(\left(V\setminus C_{1}\right)\cup \left(V\setminus C_{2}\right),C_{1}\cap C_{2}\right)+\lambda \left(\left(V\setminus C_{1}\right)\cap \left(V\setminus C_{2}\right),C_{1}\cup C_{2}\right). \end{array}$$

Set $B_{1}=V\setminus C_{1}$ and $B_{2}=V\setminus C_{2}$. The above inequality can be expressed as

$$\lambda \left(B_{1},C_{1}\right)+\lambda \left(B_{2},C_{2}\right)\geq \lambda \left(B_{1}\cup B_{2},C_{1}\cap C_{2}\right)+\lambda \left(B_{1}\cap B_{2},C_{1}\cup C_{2}\right),$$

which precisely matches the bisubmodularity condition from (23). □

## Appendix F. Proof of Proposition 5

**Proof.** Equation (31) gives (32) as an upper bound by removing the constraints for B⊉S. Equation (31) gives (33) as an upper bound by removing the constraints for B∩A≠∅. Removing both sets of constraints give (34). The polynomial time complexity follow from the submodularity of the cut in Proposition 4. In particular, the optimal direction can also be computed in polynomial-time as explained in [34]. □
